# Effectiveness of Scotland's National Naloxone Programme for reducing opioid‐related deaths: a before (2006–10) versus after (2011–13) comparison

**DOI:** 10.1111/add.13265

**Published:** 2016-02-04

**Authors:** Sheila M. Bird, Andrew McAuley, Samantha Perry, Carole Hunter

**Affiliations:** ^1^ MRC Biostatistics UnitCambridgeUK; ^2^ Department of Mathematics and StatisticsStrathclyde UniversityGlasgowUK; ^3^ NHS Health ScotlandPublic Health Science DirectorateGlasgowUK; ^4^ Institute for Applied Health Research, School of Health and Life SciencesGlasgow Caledonian UniversityGlasgowUK; ^5^ Emergency DepartmentWestern InfirmaryGlasgowUK; ^6^ Addiction Services for NHS Greater Glasgow and ClydePossilpark Health and Care CentreGlasgowUK

**Keywords:** Before/after policy evaluation, causality, effectiveness, national naloxone programme, opioid‐related deaths, prison release opioid‐related deaths, statistical power, take‐home naloxone

## Abstract

**Aims:**

To assess the effectiveness for Scotland's National Naloxone Programme (NNP) by comparison between 2006–10 (before) and 2011–13 (after NNP started in January 2011) and to assess cost‐effectiveness.

**Design:**

This was a pre–post evaluation of a national policy. Cost‐effectiveness was assessed by prescription costs against life‐years gained per opioid‐related death (ORD) averted.

**Setting:**

Scotland, in community settings and all prisons.

**Intervention:**

Brief training and standardized naloxone supply became available to individuals at risk of opioid overdose.

**Measurements:**

ORDs as identified by National Records of Scotland. Look‐back determined the proportion of ORDs who, in the 4 weeks before ORD, had been (i) released from prison (primary outcome) and (ii) released from prison or discharged from hospital (secondary). We report 95% confidence intervals for effectiveness in reducing the primary (and secondary) outcome in 2011–13 versus 2006–10. Prescription costs were assessed against 1 or 10 life‐years gained per averted ORD.

**Findings:**

In 2006–10, 9.8% of ORDs (193 of 1970) were in people released from prison within 4 weeks of death, whereas only 6.3% of ORDs in 2011–13 followed prison release (76 of 1212, *P* < 0.001; this represented a difference of 3.5% [95% confidence interval (CI) = 1.6–5.4%)]. This reduction in the proportion of prison release ORDs translates into 42 fewer prison release ORDs (95% CI = 19–65) during 2011–13, when 12 000 naloxone kits were issued at current prescription cost of £225 000. Scotland's secondary outcome reduced from 19.0 to 14.9%, a difference of 4.1% (95% CI = 1.4–6.7%).

**Conclusions:**

Scotland's National Naloxone Programme, which started in 2011, was associated with a 36% reduction in the proportion of opioid‐related deaths that occurred in the 4 weeks following release from prison.

## Introduction

Opioid overdose is a major cause of premature mortality 1. In Scotland, drugs‐related deaths (DRDs) averaged 500 per annum during 2006–10, nearly 80% of them opioid‐related 2, 3. Scotland has one of the European Union's highest DRD rates at 94 per million of population, and almost as high as the United States at 116 DRDs per million of population 4.

One‐third of receptions into Scottish prison custody test positive for opiates 5, and surveillance shows that in 2010 6, as in the 1990s, one‐third of inmates has a history of injection drug use. Scotland has some 60 000 problem drug users (PDUs) 7, 8, of whom two‐fifths are aged 35+ years. More than 20 000 clients receive opioid‐substitution therapy 5, 9, which is continued in Scottish prison custody 10.

The risk of DRD is particularly high for problem drug users after periods of relative abstinence, most notably soon after prison release 11, 12, 13, but also after hospital discharge 14, 15. The most probable reason is loss of tolerance of opioids, but prison release and hospital discharge may also mark transitions to vulnerability for other reasons. The first quantification of the high risk of DRD in the 2 weeks after prison release was made in Scotland for HIV‐infected injectors released from Edinburgh Prison in 1983–94 12. Bird & Hutchinson 13 showed that a seven times higher DRD risk in the first fortnight persisted for male releases from Scottish prison custody in 1996–99. International meta‐analysis 11 has demonstrated that prisoners' DRD risk is still elevated in the second 2 weeks post‐release. During 1996–10, White *et al*. 15 estimated 2.4 DRDs in the subsequent 4 weeks per 1000 hospital discharges of Scotland's drug treatment clients with a history of injection drug use, approximately half the DRD risk for ever‐injectors in the 4 weeks after prison release 16.

Bird & Hutchinson 13 had proposed a prison‐based randomized controlled trial of naloxone‐on‐release but it was not until 2005 that the opioid antagonist naloxone was added to the United Kingdom's exempt list of prescription‐only medicines that could be administered intramuscularly by anyone in an emergency to save life 17. Some long‐established naloxone services in America 18, 19, 20, 21, 22, 23 gave a lead, and several editorials were published 24, 25, 26. Naloxone is now provided for at‐risk patients and other first responders 1 by selected services in several European countries, Australian states and Canadian provinces.

In 2008, the UK Medical Research Council funded the pilot phase of the individually randomized N‐ALIVE Trial to test the effectiveness of naloxone‐on‐release for reducing eligible prisoners' DRDs within 4 weeks of their prison release (by 30%) and during the next 8 weeks (by 20%) 16. The N‐ALIVE Trial had been due to begin in Scotland's adult prisons but, in January 2011, was pre‐empted when Scotland became the first country internationally to introduce a centrally funded, coordinated and evaluated National Naloxone Policy (NNP) 27, 28; see Supporting information.

Across Scotland, there is a standardized naloxone‐supply scheme, and a national Patient Group Directive for supplying take‐home naloxone 29. For lay administration to adults, Scotland followed the British National Formulary recommendation of ‘intramuscular injection of 400 micrograms repeated at intervals of 2–3 minutes (in subsequent resuscitation cycles if patient not breathing normally) until consciousness regained, breathing normally, medical assistance available, or contents of syringe used up'. After completing a brief intervention (10–15 minutes), individuals at risk of opioid overdose can be prescribed naloxone. Training is delivered by a range of staff: nurses, pharmacists, voluntary‐sector workers and peer trainers, and takes place typically in community substance misuse services and pharmacies. All 15 prisons in Scotland offer naloxone‐on‐release.

Evidence of naloxone's effectiveness in reducing fatalities from opioid overdose was rated as weak by the World Health Organization 30, and has been insufficient for policy change in America 4. Scotland's NNP makes it uniquely placed to address these important limitations on empirical knowledge about naloxone's effectiveness at a population level. In this paper, we: (1) summarize the power of Scotland's before/after evaluation of its National Naloxone Policy based on well‐defined primary and secondary outcomes; (2) appraise the evidence for NNP's effectiveness by comparing outcomes in the 5 years before NNP (2006–10) and in NNP's first 3 years (2011–13); (3) apply Hill's criteria for the assessment of causality; and (4) estimate the prescription cost per quality‐adjusted life‐year (QALY) gained by ORD prevention, alternatively for 1 or 10 years.

## Methods

The N‐ALIVE team highlighted that ORDs in Scotland had been on a rising trajectory for the past decade and were therefore not optimal for evaluation purposes 28 (see also Supporting information). Instead, Scotland's evaluation should focus on those at highest risk of ORD, who might be expected to benefit most from NNP, and on modest effect sizes. The percentage of ORDs with a 4‐week antecedent of prison release (hereafter prison release ORDs) was therefore agreed as the primary outcome, with before/after evaluation periods of at least 3 years [28,31,32]. In 2012, a secondary outcome (the proportion of ORDs with a 4‐week antecedent of prison release or hospital discharge) was included to maintain statistical power at 3 years for the lower target reduction of 20%.

Pre‐NNP, 10% of ORDs were prison release ORDs. The 5‐year baseline period (2006–10) gave a pre‐NNP denominator of around 2000 ORDs. For 80% statistical power, the NNP evaluation periods were defined as follows: (a) 2011–13 (expected denominator: 1200 ORDs) to discern upper target of 30% reduction in prison release ORDs from 10 to 7%; (b) 2011–15 (expected denominator: 2000 ORDs) to discern lower target of 20% reduction in prison release ORDs from 10 to 8%; and (c) 2011–13 to discern 20% reduction in the secondary outcome 14, 15, 28 from 20 to 16%.

### Ascertainment of Scotland's primary outcome: prison release ORDs

In August of each year, National Records of Scotland releases official statistics on the number of DRDs (using the UK's harmonized definition) that were registered in Scotland in the preceding calendar year. With rare exceptions, the calendar year in which a DRD is registered in Scotland is the calendar year in which the death occurred 2, 33.

On request of the National Naloxone Advisory Group, National Records of Scotland classified DRDs in which heroin/morphine, methadone or buprenorphine was implicated in the cause of death as ORDs 3, 31, 32. To ascertain the most recent prison release date and most recent hospital discharge date for each ORD (pre‐NNP and in 2011–13), a series of permissions was obtained by Information Services Division, Scotland for its named staff to conduct the necessary look‐backs using electronically held Scottish prisoner and morbidity records, namely: from Scotland's Privacy Access Committee (twice), Scottish Prison Service and Disclosure Scotland clearances. Consequently, only in February 2012 did the National Naloxone Advisory Group learn that the pre‐NNP proportion of ORDs with a 4‐week antecedent of prison release was nearer 10% [28,31] than the expected 17% (based on extrapolation from male releases aged 15–35 years in 1996–99 13). Subsequent record‐linkage showed the pre‐NNP proportion of prison release or hospital discharge ORDs was approximately one‐fifth, with little overlap between ORDs with the two types of antecedent 3.

The 4‐week prison release antecedent counted the date of prison release as day 1, as in Bird & Hutchinson 13, but to avoid deaths in hospital the second 4‐week count started on the day after hospital discharge, as in Merrall *et al*. 14.

### Statistical methods

As designed, we use χ^2^ tests on one degree of freedom to compare the proportion of prison release ORDs between 2006 and 2010, as baseline, and 2011–13, the first 3 years of Scotland's NNP; and similarly for prison release or hospital discharge ORDs 28. We also compute 95% confidence intervals (CI) for the reduction in primary and secondary outcomes.

We define primary effectiveness as: 1 minus [proportion of ORDs in 2011–13 with 4‐week antecedent of prison release/proportion of ORDs in 2006–10 with 4‐week antecedent of prison release]. By randomly sampling (1000 times) from the independent normal distributions for the two proportions, we can compute a 95% confidence interval for NNP‐associated effectiveness in reducing prison release ORDs. Similar effectiveness calculations are made for Scotland's secondary outcome.

Scotland's before/after evaluation was non‐randomized, as Ministers had already decided that Scotland's NNP would come into effect from 2011 when advice was given on the choice of primary outcome. We therefore apply Hill's criteria 34 to appraise whether the observed reduction in prison release ORDs can be attributed to NNP.

By estimating how many fewer prison release ORDs there were during 2011–13 when Scotland issued nearly 12 000 naloxone kits, applying quality‐of‐life as only 0.7 for those at risk of ORD 35, 36 and discounting future life‐years by 3% per annum, we provide alternative 95% CIs for prescription‐cost per QALY gained if the life‐years gained by ORD prevention are 1 or 10 years.

## Results

To the nearest hundred, Scotland issued 2500 naloxone kits in 2011, 3900 in 2012 and 5500 in 2013; see Table 1. Table 1 shows that 193 (9.8%) of 1970 ORDs during 2006–10 were released from prison in the 4 weeks prior to death, but only 76 (6.3%) of 1212 ORDs in 2011–13 were χ^2^_(1)_ of 12.06, *P* < 0.001. The observed reduction in Scotland's percentage of prison release ORDs is 3.5% (95% CI = 1.6–5.4%), so that the first 3 years of Scotland's NNP are associated with a 36% reduction in the proportion of prison release ORDs (95% CI for NNP effectiveness in reducing prison release ORDs: 20–51%). These results are consistent by gender and age groups; see Table 2.

**Table 1:** Naloxone kits issued together with primary and secondary outcomes for Scotland’s National Naloxone Programme (NNP) in 2011–13.

*Period*	*Number of naloxone kits issued by*		*Number and percentage of* *Scotland*’*s opioid related deaths (ORDs) with 4‐week antecedent of:*			
	*Community and prisons*	*Prisons only*	*Prison release*		*Prison or hospital discharge*	
			*Primary outcome*		*Secondary outcome*	
2011	2487	570	36/430	8.4%	75/430	17.4%
2012	3878	725	22/399	5.5%	49/399	12.3%
2013	5533	978	18/383	4.7%	57/383	14.9%
2011–13: National Naloxone Programme	11 898	2273	76/1212	6.3%	181/1212	14.9%
					
2006–10: pre‐NNP baseline			193/1970	9.8%	374/1970	19.0%
*Analysis*						
Reduction in outcomes during NNP period			3.6%		4.1%	
95% confidence interval (CI) for reduction in outcomes during NNP period			1.6–5.4%		1.4–6.7%	
95% CI for averted prison release ORDs or secondary ORDs during NNP period			19–65		17–81	
			Averted prison release ORDs		Prison or hospital discharge ORDs	
NNP‐linked % effectiveness (computed as reduction/baseline)			36%		22%	
95% confidence interval (CI) for % effectiveness			20–51%		7–33%	

ORD = opioid‐related death.

**Table 2:** The percentage of opioid related deaths (ORDs) with prison release as 4‐week antecedent by subgroup.

*Subgroup*	*5‐year baseline: 2006–10*			*National Naloxone Programme:* *2011–13*		
	*ORDs*	*Number ORDs with 4‐week antecedent of prison release*	*% prison release ORDS*	*ORDs*	*Number ORDs with 4 week antecedent of prison release*	*% prison release ORDS*
Total	1970	193	9.8%	1212	76	6.3%
						
< 35 years	1040	126	12.1%	468	47	10.0%
35+ years	930	67	7.2%	744	29	3.9%
						
Male	1594	178	11.2%	922	65	7.0%
Female	376	15	4.0%	290	11	3.8%

**Table 3:** Age‐related increase in opioid related deaths (ORDs) by calendar year of occurrence for England (2006–13) 39 and for Scotland (2006–13) 3.

*ORDs by age group*	*2006*	*2007*	*2008*	*2009*	*2010*	*Mean per‐annum number of ORDs in 2006–10*	*SD for per‐annum ORDs in 2006–10*	*2011*	*2012*	2013
Scotland										
< 35 years	191	201	247	211	190	208.0	23.4	185	158	125
35+ years	137	169	198	221	205	186.0	33.2	245	241	258
England										
< 35 years	509	510	458	462	342	456.2	68.5	267	264	293+
35+ years	470	616	583	659	540	573.6	72.5	650	598	737+

Due to late registration of deaths in England, ORD totals for 2013 are approximately 95% complete.

During 2006–10, 19.0% of Scotland's ORDs had been released from prison or discharged from hospital in the 4 weeks prior to death versus only 14.9% in 2011–13 (χ^2^_(1)_ of 8.55, *P* = 0.003). This translates to a 22% decrease in the proportion of Scotland's ORDs with a 4‐week antecedent of prison release or hospital discharge (95% CI for NNP‐effectiveness in reducing prison release or hospital discharge ORDs: 7–33%).

Having issued nearly 12 000 naloxone kits during 2011–13, Scotland's NNP may have prevented 42 prison release ORDs (95% CI = 19–65) at a prescription cost (currently) of less than £225 000. By assuming that quality of life is only 0.7 for those at risk of ORD 35, 36 and that the life‐years gained by ORD prevention are 1 or 10 years, alternative 95% CIs for the prescription cost per QALY gained are £4900–16 900 and £560–1940 (with 3% per annum discounting if 10 life‐years were gained, QALYs would be 6.1).

### Hill's criteria for attribution of causality

Based on our application to Scotland's NNP of Hill's criteria on causality (see Supporting information), we suggest that the following are met: strength, consistency, specificity, analogy, temporality (partially), biological gradient, plausibility, coherence and experiment (partially). Here, we mention only specificity and temporality, as their justification is more intricate.

For specificity, we consider what percentage of Scotland's ORDs with a 12‐week antecedent of prison release occurred during the first 4 weeks [3], and compare this 4‐week spike‐percentage before and after the start of Scotland's NNP. Pre‐NNP, the spike‐percentage in 2006–10 was 193 of 265 (73%), but during 2011–13 it reduced significantly to 76 of 136 (56%), *P* < 0.001.

By contrast, the 4‐week spike‐percentage before and after prison‐based opioid‐substitution therapy became Scotland's health‐care standard 10 was 75% (88 of 117 ORDs) in 2000–02 (before) and unchanged thereafter at 77% (102 of 132) in 2003–07. We suggest that the change now observed in the 4‐week spike‐percentage with Scotland's NNP is specific, because a corresponding analysis for prison‐based opioid substitution therapy evinced no such reduction, either in Scotland or New South Wales 37.

For temporality, information was unavailable on England's proportion of ORDs in 2011 and 2012 with a 4‐week antecedent of prison release 38. Moreover, Table 3 shows a different pattern of ORDs by age group between England and Scotland even pre‐NNP. We note a marked decrease across 2011–13 in Scotland's younger ORDs, which is not explained by Scotland's decrease in problem drug users younger than 35 years for whom central estimates were 34 200 7 pre‐NNP and 31 000 8 currently.

A possible source of temporal confounding is the Scottish Government's 2010 Criminal Justice and Licensing Bill, which introduced a presumption against custodial sentences of up to 3 months, and came into effect from February 2011. In the Supporting information, we consider the bill's impact on opiate‐positive receptions into, and number of liberations from, Scottish prison custody pre‐NNP and during 2011–13 when there was a significant 8% decrease in the percentage of November receptions who tested positive for opiates (down from 36 to 33%), but the mean number of liberations did not increase. Temporal confounding cannot be ruled out absolutely.

## Discussion

### Primary and secondary outcomes

Scotland's issue of naloxone kits exceeded 3600 (nine times as many as Scotland's ORDs) 28 in both 2012 and 2013, but did not achieve the threshold of 8000 per annum (20 times as many as Scotland's ORDs). Availability of naloxone kits may have been close to 8000 in 2013, as kits issued in a previous year will carry over if not lost, used or out of date.

Scotland's NNP had prison release ORDs as its primary outcome: they decreased by 36% from 9.8 to 6.3% and the secondary outcome (which includes the primary) from 19 to 14.9% (22% reduction). Both are in line with the prior plausible effectiveness‐range for naloxone of 20–30%. As Scotland's NNP accelerated (2500 kits in 2011, 3900 in 2012, 5500 in 2013), reduction in the primary outcome was enhanced approximately pro rata; see Table 1.

In contrast, Scotland's NNP has had little apparent effect on the hospital discharge component of its secondary outcome 3. The reason may be both lack of power to discern an effect size more modest than 30% on hospital discharged ORDs *per se* and that high DRD risk soon after hospital discharge is less well known by opioid users—even in Scotland—than the internationally endorsed DRD risk soon after prison release 12. Future research should aim to explore this further.

If we assume that Hill's criteria for causality 34 are mostly met (see Supporting information), naloxone's effectiveness for reducing prison release ORDs is supported with a lower 95% confidence limit of 20% effectiveness. However, generalization from effectiveness for prison release ORDs cannot be taken for granted, neither within Scotland, as the 95% CI for effectiveness on prison release or hospital discharge ORDs warns (7–33%), nor outside the United Kingdom, where prisons or communities might be less efficient in issuing naloxone kits, clients may be more or less adept in their retention and use and intrinsic ORD risk may also be different. Uncertainty about secondary effectiveness being still wide, the fifth year of Scotland's NNP provides an opportunity to encourage hospital doctors and general practitioners to prescribe naloxone kits to at‐risk clients on discharge from any hospital stay.

### Cost‐effectiveness

Detailed cost‐effectiveness depends upon how many QALYs the averting of one prison release ORD represents (illustrated for the range 0.7–6.1), the non‐prescription costs associated with naloxone (including outreach and brief training) and any averted or additional costs (fewer presentations to accident and emergency; extra criminal justice costs). There may be additional averted ORDs which neither Scotland's primary outcome nor our outline cost‐effectiveness counts; for example, some of the observed decrease in ORDs in the younger age group. It is unlikely that any of these considerations—apart from criminal justice costs—would deny naloxone's cost‐effectiveness; see also Coffin *et al*. 40. Excessive prescription or outreach costs which exceeded half the current prescription cost (£19) should, however, be avoided.

### Study implications

Our findings have important implications for public health authorities across the United Kingdom and internationally. Wales, like Scotland, has a NNP. Unlike Scotland, Wales (41, 42 and personal communication from Dr Josie Smith, *Harm Reduction Database Wales*: *Take Home Naloxone 2013–2014,* Public Health Wales, 15 December 2014) does not have sufficient ORDs (approximately 85 per annum pre‐NNP) for a 5‐year before/after evaluation of its NNP to be statistically powerful.

England has yet to formalize a monitored NNP [43,44] but would need to issue a minimum of 9000 naloxone kits annually, ideally 20 000 28. With 1000 ORDs per annum in England, the impact of an efficiently implemented NNP could be evident as a marked reduction in England's prison release ORDs within 18 months [39] when compared with the previous 30 months.

In 2014, the N‐ALIVE pilot trial randomized 80 eligible prisoners per month in English prisons who, at liberation, received an N‐ALIVE pack which either contained naloxone for intramuscular injection or did not. In the light of Scotland's 3‐year results and N‐ALIVE's own data on ex‐prisoners' altruistic administration, N‐ALIVE's Trial Steering and Data Monitoring Committee agreed that an individually randomized main trial was unfeasible and that randomization should cease with not‐yet‐released participants being offered naloxone‐on‐release. This decision was implemented on 8 December 2014^1^ and peer‐review publications will follow.

### Strengths and limitations of this study

This study is the first attempt to evaluate a national naloxone programme at a population level with before/after analyses by design at 3 years and 5 years.

Its strengths include ministerial acceptance of methodological advice that the primary outcome in Scotland's NNP evaluation should be reduction in prison release ORDs, not in ORDs which were liable to strong systematic and idiosyncratic variation; see Table 3 and Supporting information. Secondly, Scotland's evaluation was powered adequately to discern modest 20–30% effectiveness. Thirdly, the Information Services Division, tasked with deriving and publishing Scotland's primary and secondary outcomes as official statistics, is bound by the code of practice for the UK's official statisticians, which ensures freedom from political or other influence. Fourthly, ministerial acceptance of a standardized naloxone‐supply across Scotland meant that quarterly issue and reimbursement of naloxone kits could be monitored closely—not least because most clients gave consent for demographical details to be centralized. Every 2 years, there is information from Scotland's Needle Exchange Surveillance Initiative 45 about the proportion of its injector‐interviewees who were prescribed naloxone (and by whom) in the past year [28]. Fifthly, the non‐randomized before/after design for NNP's evaluation recognizes that community‐prescribed naloxone is more likely to be administered to someone other than the person for whom it was prescribed 22, 28, which compromises individually randomized studies of naloxone's beneficiaries.

Acknowledged limitations are, first, that the NNP start was determined by Ministers and was not subject to randomization; for example, as a randomized regional step‐wedge design. Confounding of Scotland's before/after NNP evaluation cannot be ruled out; hence our need to appraise causality by Hill's criteria and the recommendation that other nations' NNPs be also evaluated formally. Secondly, Scotland's evaluation periods were extended from 3 to 5 years, and a secondary outcome defined when it was identified that prison release ORDs were only 10% of Scotland's ORDs pre‐NNP [28]. Thirdly, drug treatment clients' high DRD risk in the 4 weeks after any hospital discharge, which is a key component of our secondary outcome, has been validated within Scotland (for 2006–10 15), but no study outside Scotland has yet endorsed or challenged it. Fourthly, the beneficiary of community‐issued naloxone is typically not the person for whom it was prescribed 28: NNP's beneficiaries are therefore not individually identifiable. The N‐ALIVE pilot trial ceased individual randomizations because its own feedback, together with Scotland's data 3, 31, 32, confirmed that only one‐third of naloxone‐on‐release kits are used on the ex‐prisoner for whom the kit was prescribed. Without assortative mixing, this one‐third rate is lower than would be expected if recently released prisoners' high ORD rate was due to high overdose rate rather than high fatality rate per opioid overdose. If the latter holds, then a further limitation is that NNP's effectiveness in respect of prison release ORDs could be an overestimation of its generalized effectiveness. Finally, generalization from a single well‐powered national before/after evaluation could be recommended more confidently if Scotland's results were validated elsewhere.

^1^Update for people who have taken part in the N‐ALIVE pilot trial [internet, cited 27 November 2015]. Available at: http://www.ctu.mrc.ac.UK/13391/13399/n‐alive_update_12.14.

## Conclusion

With 2 years of Scotland's National Naloxone Programme to follow, the current data suggest at least 20% and best estimate of 36% reduction in prison release ORDs, which may be due directly to the programme. Scotland's 3‐year results may encourage other nations to adopt naloxone policies. Implementation of national naloxone programmes should be monitored closely and results put into the public domain to facilitate evidence synthesis.

## Declaration of interests

S.M.B. is a co‐principal investigator for the MRC‐funded N‐ALIVE pilot Trial which, by unanimous decision of its Trial Steering and Data Monitoring Committee, ceased randomization on 8 December 2014 and offered naloxone‐on‐release to already‐randomized, not‐yet‐released prisoners who had been assigned to the control group. All four authors serve on Scotland's National Naloxone Advisory Group, which C.H. chairs, but write in a personal capacity. S.M.B. was, on two occasions, a funded invited speaker at the Academic and Health Policy Conference on Correctional Health. S.M.B. holds GSK shares.

## Ethical approval

Approval in the public interest was obtained by the NHS National Services Scotland Information Services Division from Scotland's Privacy Access Committee to conduct the look‐back from Scotland's drugs‐related deaths in 2006–15 to obtain dates of (i) most recent prison release and (ii) most recent hospital discharge. Those to whom naloxone was prescribed gave freely (or withheld) informed consent for their demographic details to be centralized for analysis by Scotland's Information Services Division.

## Supporting information




**Section 1** Application of Hill's criteria on causality.
**Section 2** Methodological difficulties in evaluating the effectiveness of lay administration of naloxone.
**Section 3** Additional information on the set‐up of Scotland's National Naloxone Policy.
**Section 4** Confirmation of assumptions about greater heterogeneity in Scotland's pre‐NNP number of ORDs than in percentage of ORDs with prison release as 4‐week antecedent.

Supporting info item
