# Supplementary material for: Effectiveness of Scotland's National Naloxone Programme for reducing opioid‐related deaths: a before (2006–10) versus after (2011–13) comparison
Source: Addiction. 2016 Feb 4;111(5):883–91. doi: 10.1111/add.13265 (PMC4982071; doi:10.1111/add.13265)
Supplement: Supplementary file 1 — Supporting info item [file ADD-111-883-s001.docx]

**SUPPLEMENTARY MATERIAL**

***Section 1****: Application of Hill’s criteria on causality*

***Strength:*** *The larger the association of primary outcome with NNP, the more likely to be causal.* Applies.

***Consistency:*** *Consistent findings - by different persons in different places and with different samples - strengthen the likelihood of an effect.* Applies for primary outcome across sub-groups by age group or gender, see **Table 2**; not proven outside of Scotland as comparable data for England and Wales are not available. Age-related increase in DRDs in 21^st^ century applies in England too, see **Table 3**.

***Specificity:*** *The more specific the association between NNP and Scotland’s primary outcome, the greater the probability that NNP is the reason for Scotland’s percentage of ORDs with 4-week prison release antecedent having decreased in 2011-2013*. Applies for pre-defined primary outcome; %-effectiveness was also pre-specified in power calculations. Reduction in the 4-week spike of prison release ORDs did not occur with prison-based opioid-substitution therapy.

***Temporality:*** *The observed decrease in primary and secondary outcomes has to occur after NNP.* Applies, *except for* some evidence of heterogeneity in the primary outcome during 2006-2010, which could suggest the existence of an unrecognised confounder. For example, the Scottish Government’s 2010 Criminal Justice and Licensing Bill introduced a presumption against custodial sentences of up to three months. Since the change was implemented in February 2011 there has been an 8% decrease in sentences of up to two years (from mean of 14,850 in the previous four years to 13,687 in the first three implementation-years) with 14% increases in sentences of duration 3 months to 2 years (up from 8,218 to 9,361) offsetting a 35% decrease in sentences up to 3 months (down from mean of 16,120 to mean of 14,900). Scottish Prison Service’s mean number tested for drugs at reception in November of 2011 to 2013 (1305) was higher, not lower, than the mean for three preceding NNP-baseline years for which data were available (1183) and unchanged for tests on liberation (728 versus 723 earlier). Opiate positive rate at reception did reduce by 8%, however, from 36% (95% CI: 34% to 39%) to 33% (05% CI: 31% to 34%) and so some temporal confounding cannot be ruled out.

Temporal patterns in ORDs by age group, as shown in **Table 3**, were different between Scotland and England even before NNP. Data on prison release ORDs are not available for England.

***Biological gradient:*** *Greater exposure (naloxone kits issued during 2011-2013) should generally lead to greater incidence of the effect.* Applies, see **Table 1** which shows that 2012 was the first year that the minimum proposed threshold for Scotland of 3600 naloxone kits issued per annum^6^ was exceeded.

***Plausibility:*** *A plausible mechanism between NNP and primary and secondary outcomes is helpful.* Applies more to primary than to secondary outcome. The 4 weeks after prison release are recognised internationally as a period of markedly high DRD-risk, whereas the 4 weeks after hospital discharge have been validated as a period of high DRD-risk in Scotland only. Unlike Scotland’s prisons, and despite a communication from Scotland’s Chief Medical Officer in January 2011, hospitals were not specifically targeted to prescribe naloxone kits to at-risk clients.

***Coherence*:**  *Coherence between epidemiological and laboratory findings increases the likelihood of an effect.* Applies in respect of Scotland’s Needle Exchange Surveillance Initiative^61^ which suggested a 2.5 to 3 fold increase in past-year prescription of take-home naloxone to current injectors between summer 2011 and summer 2013, with most kits apparently issued to current injectors^6^. Reduction in primary outcome was 3-fold greater in 2013 than in 2011.

***Experiment:*** *“Occasionally it is possible to appeal to experimental evidence”.* Among 1600 prisoners randomized in the N-ALIVE pilot Trial, fewer than 10 DRDs are expected within 12 weeks of release, which cannot add substantially (in either direction) to the evidence-base from Scotland’s policy-evaluation. N-ALIVE pilot Trial does support Scotland’s evidence for the ratio of naloxone-on-release kits used on the prisoner for whom prescribed versus on others as being 1:2, Scotland having reported 12:21 for naloxone-on-release kits but 110:626 for community-issued naloxone kits; and the N-ALIVE pilot Trial 5:15, see http://www.ctu.mrc.ac.uk/our_research/research_areas/other_conditions/studies/n_alive/.

***Analogy:*** *The effect of similar factors may be considered.* Similar factor is the impact of Scotland’s prison-based opioid-substitution therapy (as healthcare standard) on the spike of ORDs soon after release, see **Results**. Another aspect is to consider what was happening to ORDs elsewhere in the UK, see **Table 3**.

***Section 2****: Methodological difficulties in evaluating the effectiveness of lay administration of naloxone*

Evidence about naloxone’s effectiveness in reducing fatalities from opioid overdose was rated weak by WHO^30^ and has been insufficient for policy-change in America^4^.

Methodological difficulties include an age-related rise in opioid-related deaths (ORDs) in the 21^st^ century ^28^ (see **Table 3**) which compounds idiosyncratic year-on-year changes in the number of ORDs, confounding their before/after comparison. Secondly, regression-to-mean bias occurs if before-locations are specifically chosen because of high DRD-rates (see Opioid overdose rates and implementation of overdose education and nasal naloxone distribution in Massachusetts: interrupted time series analysis. *British Medical Journal*, 2013; 346: f174 doi: 10.1136/bmj.f174).

Thirdly, effective lay administration requires an opioid overdose to be witnessed, naloxone to have been carried (by victim or witness), the witness to administer it, and to have done so with a timeliness unmatchable by the subsequent arrival of ambulance staff ^16 28^. Given that the survival-rate is generally high at witnessed opioid overdoses^28^, studies of take-home naloxone have consequently lacked statistical power to discern modest effectiveness^16 39^.

Finally, the N-ALIVE pilot Trial^16^ concluded in December 2014 that a prison-based, individually-randomized controlled trial was not a feasible methodology because, in the majority of cases, naloxone-on-release is administered altruistically to others, twice as often as to the ex-prisoner for whom it was prescribed. Take-home naloxone, as prescribed by community services, is even less likely to be administered to its recipient ^3 28 22^.

These methodological difficulties make an adequately-powered policy-evaluation the best option: using a randomized wedge-design; or before/after comparison with a more subtle primary outcome than number of ORDs. Scotland chose the latter. Please see our **Discussion** (*Strengths and limitations of this study*) and ***Section 3*** for an account of how Scotland overcame methodological difficulties.

***Section 3****: Set-up of Scotland’s National Naloxone Policy*

The Scottish Government made £400,000 available for the reimbursement of 33,000 naloxone kits prescribed to (or on behalf of) users who had a history of opioid dependency: 6,000 naloxone kits annually in the community and 5,000 to Scotland’s eligible prisoners on-release. See *Tackling Drug Deaths,* the Scottish Government announcement on 17 August 2010. (see <http://www.scotland.gov.uk/News/Releases/2010/08/17095105>. Archived at http://www.webcitation.org/6amQ9wOmt on August 14^th^ 2015).

Initially, naloxone kits cost £12, currently £19. Scotland appointed a national naloxone co-ordinator at Scottish Drugs Forum to cascade training. There was specific support for the Scottish Prison Service, and national naloxone information materials were developed. See **FIGURE** for initial tasks.

Individuals with a current or historic use of opioids who are at-risk of ORD are eligible for naloxone. Addiction services or harm reduction teams are responsible for issuing the majority of naloxone kits in the community but some community-pharmacies and peer-to-peer delivery of brief naloxone training contribute also. At-risk clients are offered naloxone training as a 10-15 minute brief intervention, after which their naloxone-kit is supplied via the national Patient Group Directive.

All Scottish Prison Service’s establishments, whether public or private, offer naloxone-on-release. Eligible prisoners are identified as at-risk of ORD during their initial healthcare assessment or subsequently; and, close to liberation or court date, are offered a place on one of the prison’s naloxone training sessions. Following successful completion of training, a naloxone-kit is placed within the prisoner’s property-valuables, for receipt upon liberation.

The National Naloxone Advisory Group proposed targets in the financial-year 2013/14 for the community issue of naloxone kits to ensure that during 2011/12 to 2013/14 each health board’s issue of naloxone kits should exceed 15% of its estimated regional number of problem drug users^7^. The target of 9,000 community-issued naloxone kits across Scotland was duly achieved^3^. By the end of 2014/15, the community-issue of naloxone kits should exceed 25% of a region’s estimated number of problem drug users; or 15,000 across Scotland. Targets for individual prisons were first set for 2014/15.

**FIGURE:** A dozen tasks in the set-up or evaluation of Scotland’s National Naloxone Policy.

1. With-consent database on the demography of those to whom naloxone kits were issued in the community or by prisons as first supply or re-supply: created by Information Services Division (ISD) under their contractual arrangement with Scotland’s Drug Policy Unit. To ensure a high consent-rate, ISD gave an undertaking that there would be no linkage of the naloxone-database to any other, not even to the deaths-register.
2. For those who are re-supplied, the above database records answers to questions about what happened to the previously-supplied naloxone-kit and in particular, if naloxone was administered, was the beneficiary the named patient or another person.
3. Purchase-price for, and provider of, Scotland’s naloxone kits: supplied by Martindale Pharma (prefilled syringe containing 2mls of 1mg/1ml naloxone hydrochloride). Two needles for intramuscular injection were added, together with a tailored patient information leaflet and basic first aid advice. The kit was assembled and resealed by Tayside Pharmaceuticals who held an enabling licence from the Medicines and Healthcare Regulatory Authority. The reassembled product cost £12 and was used until 2013, when Martindale Pharma introduced and Scotland adopted Prenoxad®, as the first licensed product designed for the emergency treatment of opioid overdose by lay persons in non-medical settings. Prenoxad® contains one prefilled syringe of 2mls of 1mg/1ml naloxone hydrochloride, two 23G * 1.25 inch needles and a patient information leaflet; and costs £18.90 inclusive of taxes.
4. Quarterly notification to ISD of the number of naloxone kits purchased by each regional health authority in Scotland; and by Scotland’s prisons.
5. In October 2010, NNAG formally defined opioid-related deaths (ORDs) as DRDs in which heroin/morphine, methadone or buprenorphine was implicated in the cause of death and established Scotland’s baseline period as 2007-2009 (later 2006-2010). One in six of around 1200 ORDs in 2007-2009 was expected to have a 4-week antecedent of prison release.
6. Drugs-related deaths (DRDs) registered in 2007-2009 were identified to ISD by National Records of Scotland with an indicator variable which categorized each as ORD or not.
7. Under the contractual arrangement between ISD and Scotland’s Drugs Policy Unit, ISD was tasked with establishing - via unique-prisoner-number databases held by Scottish Prison Service - for each DRD and, especially for each ORD, whether the deceased had ever been incarcerated in Scotland; and, if so, date and prison of last-release, and whether the release had been from court or from prison (release-date equals warrant-date for those released from court).
8. The above record-linkage, which enabled ISD, on behalf of NNAG, to compute the percentage of ORDs with 4-week (or 12-week) antecedent of prison release, required that an application was made by ISD to Scotland’s Privacy Access Committee. The application restricted the conduct of the look-back to be carried through by ISD-employed staff.
9. Criminal Record Bureau clearance was required before ISD staff could access the antecedent Scottish incarceration histories of ORDs.
10. Attempts were made to collate annual reports on call-outs to Scottish Ambulance Service to attend opioid-overdoses; and on the number of overdose victims who were attended by paramedical teams and hospitalized as a consequence; but floundered.
11. For the purpose of setting targets for the number of naloxone kits to be issued per prison, NNAG accessed official statistics on a prison’s recent number of liberations per annum and, from Scottish Prison Service, the proportion of a prison’s receptions into custody who tested positive for opiates in three surveillance-years.
12. Peer-trainers, including in prisons, began work in 2012**,** having been trained to deliver brief interventions by a National Peer Trainer who works alongside the National Naloxone Co-ordinator. Peer-trainers are mentored for at least 6-months after their initial training.

***Section 4****: Confirmation of heterogeneity assumptions*

As anticipated by the N-ALIVE team’s statistical advice, the heterogeneity in Scotland’s number of ORDs across 2006-2010 was greater than in either the percentage of ORDs with prison release as 4-week antecedent (**Table S1**)or the percentage with hospital discharge as 4-week antecedent (which is a constituent-part of the secondary outcome, see **Table S2**): chi-square statistics on 4df of 21.3 (p = 0.0003), 11.3 (p = 0.023), and 5.8 (p = 0.22) respectively.

**Table S1:** Heterogeneity during 2006-2010 in the number of opioid-related deaths (ORDs) and in the percentage of ORDs with prison release as 4-week antecedent^3^.

| **YEAR** | **Five calendar years before Scotland’s**  **National Naloxone Programme (NNP)** | | | | | | **First three calendar years of Scotland’s NNP** | | | |
| --- | --- | --- | --- | --- | --- | --- | --- | --- | --- | --- |
|  | *Prison release ORDs (as percentage of calendar year ORDs)* | | | | | | | | | |
|  | 2006 | 2007 | 2008 | 2009 | 2010 | **5-year**  **Total** | 2011 | 2012 | 2013 | **3-year**  **Total** |
| *Prison release*  ORDs | *35 (11%)* | *40*  *(11%)* | *56*  *(13%)* | *38*  *( 9%)* | *24*  *( 6%)* | ***193***  ***(9.8%)*** | *36*  *(8.4%)* | *22 (5.5%)* | *18*  *(4.7%)* | ***76***  ***(6.3%)*** |
| Other ORDs | 293 | 330 | 389 | 394 | 371 | **1777** | 394 | 377 | 365 | **1136** |
| **Totals** | 328 | 370 | 445 | 432 | 395 | **1970** | 430 | 399 | 383 | **1212** |

**Table S2:** Heterogeneity during 2006-2010 in the percentage of ORDs with hospital discharge as 4-week antecedent^3^.

| **YEAR** | **Five calendar years before Scotland’s**  **National Naloxone Programme (NNP)** | | | | | | **First three calendar years of Scotland’s NNP** | | | |
| --- | --- | --- | --- | --- | --- | --- | --- | --- | --- | --- |
|  | *Number of Hospital discharge ORDs (also as percentage of each year’s ORDs)* | | | | | | | | | |
|  | 2006 | 2007 | 2008 | 2009 | 2010 | **5-year**  **Total** | 2011 | 2012 | 2013 | **3-year**  **Total** |
| *Hospital discharge*  ORDs | *22*  *( 7%)* | *33*  *( 9%)* | *51*  *(11%)* | *42*  *(10%)* | *43*  *( 11%)* | ***191***  ***(9.7%)*** | *43*  *(10%)* | *28*  *( 7%)* | *40*  *(10%)* | ***111***  ***(9.1)*** |
| Other ORDs | 306 | 337 | 394 | 390 | 352 | **1779** | 387 | 371 | 343 | **1101** |
| **Totals** | 328 | 370 | 445 | 432 | 395 | **1970** | 430 | 399 | 383 | **1212** |
